# [*N*-Benzyl-*N*-(2-phenyl­eth­yl)di­thio­carbamato-κ^2^ *S*,*S*′]tri­phenyl­tin(IV) and [bis­(2-meth­oxy­eth­yl)di­thio­carbamato-κ^2^ *S*,*S*′]tri­phenyl­tin(IV): crystal structures and Hirshfeld surface analysis

**DOI:** 10.1107/S2056989016014985

**Published:** 2016-09-27

**Authors:** Rapidah Mohamad, Normah Awang, Nurul Farahana Kamaludin, Mukesh M. Jotani, Edward R. T. Tiekink

**Affiliations:** a Biomedical Science Programme, School of Diagnostic and Applied Health Sciences, Faculty of Health Sciences, Universiti Kebangsaan Malaysia, Jalan Raja Muda Abdul Aziz, 50300 Kuala Lumpur, Malaysia; b Environmental Health and Industrial Safety Programme, School of Diagnostic and Applied Health Sciences, Faculty of Health Sciences, Universiti Kebangsaan Malaysia, Jalan Raja Muda Abdul Aziz, 50300 Kuala Lumpur, Malaysia; c Department of Physics, Bhavan’s Sheth R. A. College of Science, Ahmedabad, Gujarat 380001, India; d Research Centre for Chemical Crystallography, Faculty of Science and Technology, Sunway University, 47500 Bandar Sunway, Selangor Darul Ehsan, Malaysia

**Keywords:** crystal structure, organotin, di­thio­carbamate, Hirshfeld surface analysis

## Abstract

The crystal and mol­ecular structures of two tri­phenyl­tin di­thio­carbamates, [Sn(C_6_H_5_)_3_(C_16_H_16_NS_2_)], (I), and [Sn(C_6_H_5_)_3_(C_7_H_14_NO_2_S_2_)], (II), are described. In (I), the di­thio­carbamate ligand coordinates the Sn^IV^ atom in an asymmetric manner, leading to a highly distorted trigonal–bipyramidal coordination geometry defined by a C_3_S_2_ donor set with the weakly bound S atom approximately *trans* to one of the *ipso*-C atoms. A similar structure is found in (II), but the di­thio­carbamate ligand coordinates in an even more asymmetric fashion. The packing in (I) features supra­molecular chains along the *c* axis sustained by C—H⋯π inter­actions; chains pack with no directional inter­actions between them. In (II), supra­molecular layers are formed, similarly sustained by C—H⋯π inter­actions; these stack along the *b* axis. An analysis of the Hirshfeld surfaces for (I) and (II) confirms the presence of the C—H⋯π inter­actions but also reveals the overall dominance of H⋯H contacts in the respective crystals.

## Abstract 2

Heavily distorted trigonal–pyramidal coordination geometries, each based on a C_3_S_2_ donor set and with the loosely bound S atom approximately *trans* to one of the *ipso*-C atoms, are found in the title compounds (C_6_H_5_)_3_Sn[S_2_CN(Ben)CH_2_CH_2_Ph] and (C_6_H_5_)_3_Sn[S_2_CN(CH_2_CH_2_OMe)_2_].

## Chemical context   

Among the varied motivations for investigating organotin di­thio­carbamate compounds, *i.e. R_n_*Sn(S_2_CN*RR*′)_4–*n*_ where *R*, *R*′ = alkyl, aryl, most relate to their biological activities and their usefulness as mol­ecular, single-source precursors for the formation of tin sulfide nanoparticles (Tiekink, 2008 ▸). In terms of the latter, while triorganotin di­thio­carbamates, *i.e*. with *n* = 3, have been examined in this context (Kana *et al.*, 2001 ▸), di- and mono-organotin derivatives often provide more effective precursors (Ramasamy *et al.*, 2013 ▸). By contrast, significant inter­est in the biological effects of triorganotin di­thio­carbamates continues. Hence, a wide variety of biological applications of triorganotin di­thio­carbamates, *i.e*. directly related to the title compounds, have been investigated. Thus, anti-bacterial (Muthalib *et al.*, 2015 ▸), larvicidal (Song *et al.*, 2004 ▸), including against mosquito larvae (Basu Baul *et al.*, 2005 ▸), insecticidal (Awang *et al.*, 2012 ▸; Safari *et al.*, 2013 ▸) and anti-leishmanial activities (Ali *et al.*, 2014 ▸) have been investigated. However, most activity has been directed towards evaluating their potential as anti-cancer agents (Tiekink, 2008 ▸; Khan *et al.*, 2014 ▸, 2015 ▸). It was in this context and during on-going structural studies of organotin di­thio­carbamates (Muthalib *et al.*, 2014 ▸; Mohamad *et al.*, 2016 ▸) that the title compounds were synthesized. Herein, the crystal and mol­ecular structures of (C_6_H_5_)_3_Sn[S_2_CN(Ben)CH_2_CH_2_Ph] (I) and (C_6_H_5_)_3_Sn[S_2_CN(CH_2_CH_2_OMe)_2_] (II) are reported along with a detailed analysis of the supra­molecular association operating in their crystal structures by means of Hirshfeld surface analysis.
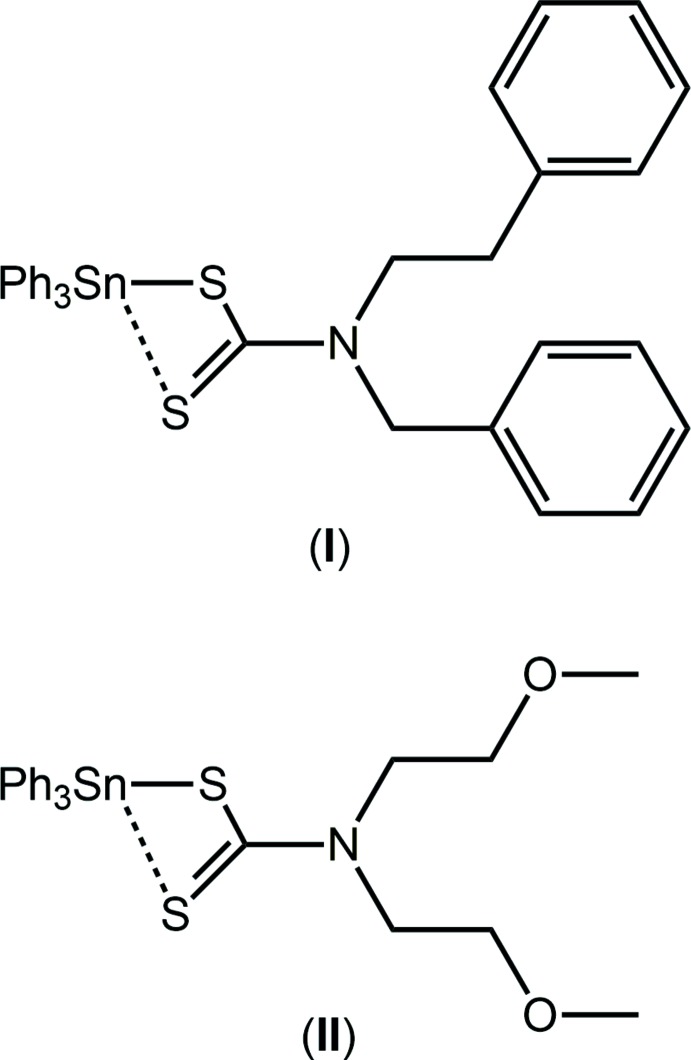


### Structural commentary   

**Figure 1:**
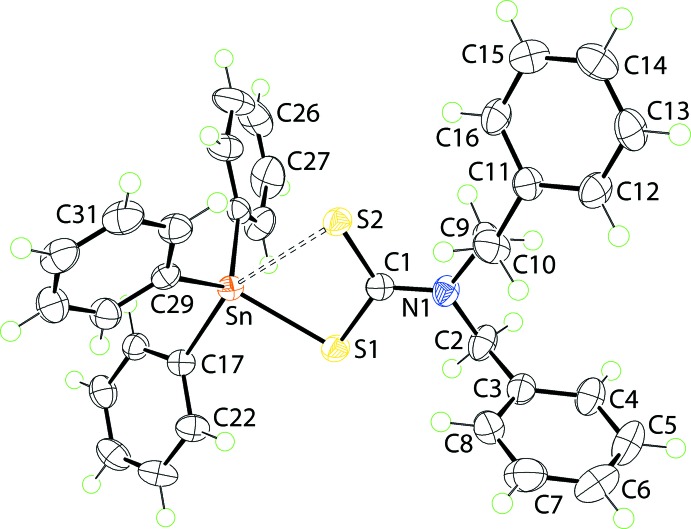
The mol­ecular structure of (I), showing the atom-labelling scheme and displacement ellipsoids at the 70% probability level.

The mol­ecular structure of (I) is shown in Fig. 1 ▸ and selected geometric parameters are collected in Table 1 ▸. The tin atom is bound to three phenyl groups and to the di­thio­carbamate ligand. The latter coordinates asymmetrically with Δ(Sn—S), being the difference between the Sn—S_long_ and Sn—S_short_ bond lengths, of 0.42 Å. This asymmetry is reflected in the relatively large disparity in the associated C—S bond lengths with the bond involving the tightly bound S1 atom being significantly longer than the bond involving the S2 atom, Table 1 ▸. Such a great difference might imply a monodentate mode of coordination for the di­thio­carbamate ligand and the adoption of a tetra­hedral coordination geometry. However, the range of tetra­hedral angles if this were the case is over 30°, *i.e*. from a narrow 92.98 (4)° for S1—Sn—C17 to a wide 124.31 (4)° for S1—Sn—C29. The wide angle is due to the close approach to the tin atom of S2. Further, the Sn—C17 bond length is systematically longer than the other Sn—C bond lengths, an observation ascribed to the C17 atom being approximately *trans* to the incoming S2 atom, Table 1 ▸. Thus, the coordination geometry is best described as being based on a C_3_S_2_ donor set. The geometry is not ideal with the value of τ of 0.57, *cf*. τ values of 0.0 and 1.0 for ideal square–pyramidal and trigonal–bipyramidal geometries, respectively (Addison *et al.*, 1984 ▸), suggesting a small distortion towards trigonal–bipyramidal. Distortions from the ideal can be related to the disparate Sn—donor atom bond lengths and the acute chelate angle, Table 1 ▸.

**Figure 2:**
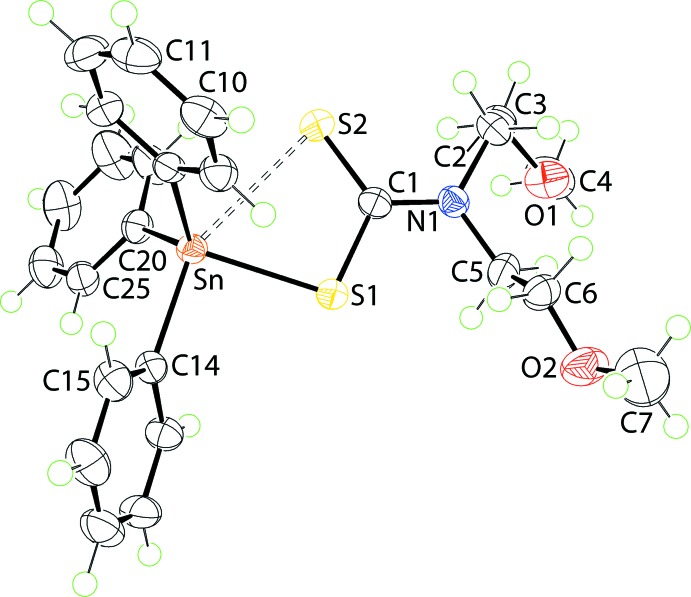
The mol­ecular structure of (II), showing the atom-labelling scheme and displacement ellipsoids at the 70% probability level. Only the major component of the disordered C5–C6–O2–C7 chain is shown, where atoms C6 and O2 are split over two positions.

The mol­ecular structure of (II) (Fig. 2 ▸) bears many similarities with that just described for (I). The value of Δ(Sn—S) of 0.64 Å is even greater than that of (I), indicating a more asymmetric mode of coordination of the di­thio­carbamate ligand. This difference is also reflected in the associated C—S bond lengths, following the same trend as for (I) but, with Δ(C—S) of 0.08 Å *cf*. 0.06 Å for (I). This being stated, the Sn—C14 bond length of 2.1608 (14)°, with the C14 atom being *trans* to the S2 atom, is the longest of all six Sn—C bonds in (I) and (II). The range of tetra­hedral angles, *i.e*. 90.94 (4)° for S1—Sn—C14 to 119.54 (5)° for C8—Sn—C20, is slightly narrower at less than 30°. The value of τ computes to 0.58, *i.e*. virtually identical to that in (I).

## Supra­molecular features   

**Figure 3:**
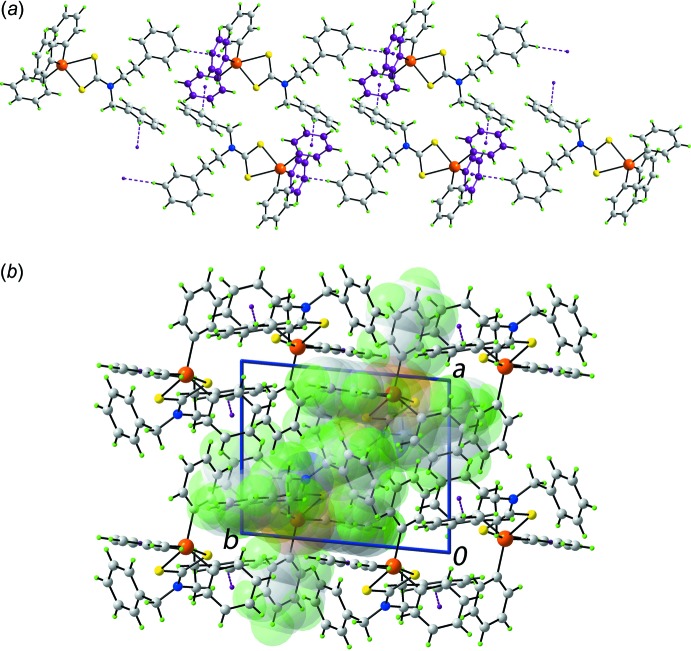
The mol­ecular packing in (I): (*a*) supra­molecular chain along the *c* axis sustained by di­thio­carbamate-phenyl-C—H⋯π(Sn-phen­yl) inter­actions shown as purple dashed lines and (*b*) a view of the unit-cell contents in projection down the *c* axis. In (*a*), the accepting rings are highlighted in purple and in (*b*), one chain is highlighted in space-filling mode.

Despite there being five aromatic rings in the mol­ecule of (I), the closest face-to-face contact between rings is > 4.0 Å. The only points of contact between mol­ecules in the mol­ecular packing identified by *PLATON* (Spek, 2009 ▸) are those of the type C—H⋯π. Each of the rings of the di­thio­carbamate ligand donates a hydrogen atom to a different tin-bound phenyl ring with the result that a supra­molecular chain is formed along the *c*-axis direction, Table 2 ▸ and Fig. 3 ▸ *a*. The chains pack without directional inter­actions between them, Fig. 3 ▸ *b*.

**Figure 4:**
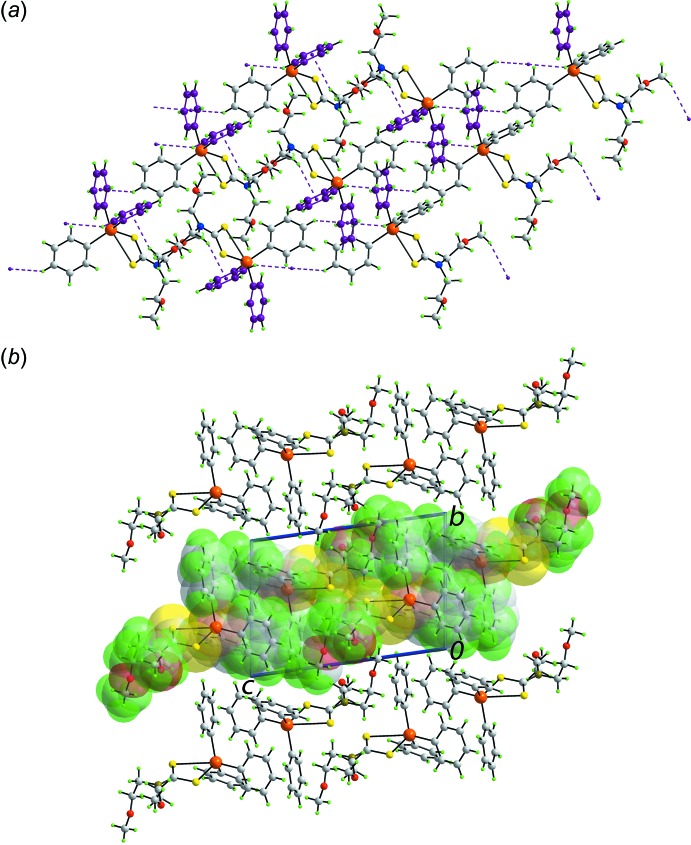
The mol­ecular packing in (II): (*a*) supra­molecular layer parallel to the *ac* plane sustained by methyl- and Sn-phenyl-C—H⋯π(Sn-phen­yl) inter­actions shown as purple dashed lines and (*b*) a view of the unit-cell contents in projection down the *a* axis. In (*a*), the accepting rings are highlighted in purple and in (*b*), one layer is highlighted in space-filling mode.

Even though there are oxygen atoms in the mol­ecule of (II), the supra­molecular association is dominated by C—H⋯π contacts involving methyl-C—H and Sn-bound-phenyl-C—H as donors and only two of the Sn-bound phenyl rings as acceptors, as the (C14–C19) ring accepts two inter­actions, Table 3 ▸. The result of this association is the formation of supra­molecular layers in the *ac* plane, Fig. 4 ▸ *a*. The layers stack along the *b* axis without directional inter­actions between them, Fig. 4 ▸ *b*.

## Hirshfeld surface analysis   

*Crystal Explorer* (Wolff *et al.*, 2012 ▸) was used to generate Hirshfeld surfaces mapped over *d*_norm_, shape-index and electrostatic potential. The electrostatic potentials were calculated using *TONTO* (Spackman *et al.*, 2008 ▸; Jayatilaka *et al.*, 2005 ▸) integrated into *Crystal Explorer*, wherein the respective experimental structure was used as the input to *TONTO*. Further, the electrostatic potentials were mapped on Hirshfeld surfaces using the STO-3G basis set at the Hartree–Fock level of theory over ranges ± 0.037 au. and ± 0.048 au. for (I) and (II), respectively. The contact distances *d*_i_ and *d*_e_ from the Hirshfeld surface to the nearest atom inside and outside, respectively, enable the analysis of the inter­molecular inter­actions through the mapping of *d*_norm_. The combination of *d*_e_ and *d*_i_ in the form of two-dimensional fingerprint plots (McKinnon *et al.*, 2007 ▸) provides a useful summary of inter­molecular contacts in the respective crystal.

**Figure 5:**
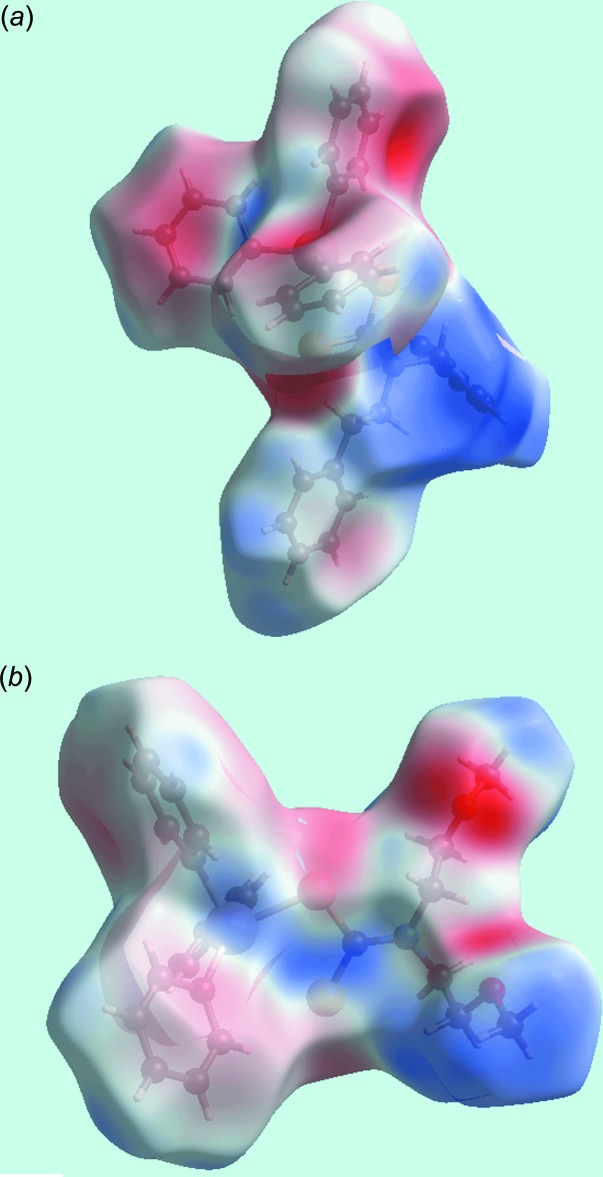
Views of the Hirshfeld surfaces mapped over electrostatic potential (the red and blue regions represent negative and positive electrostatic potentials, respectively): (*a*) for (I) and (*b*) for (II).

**Figure 6:**
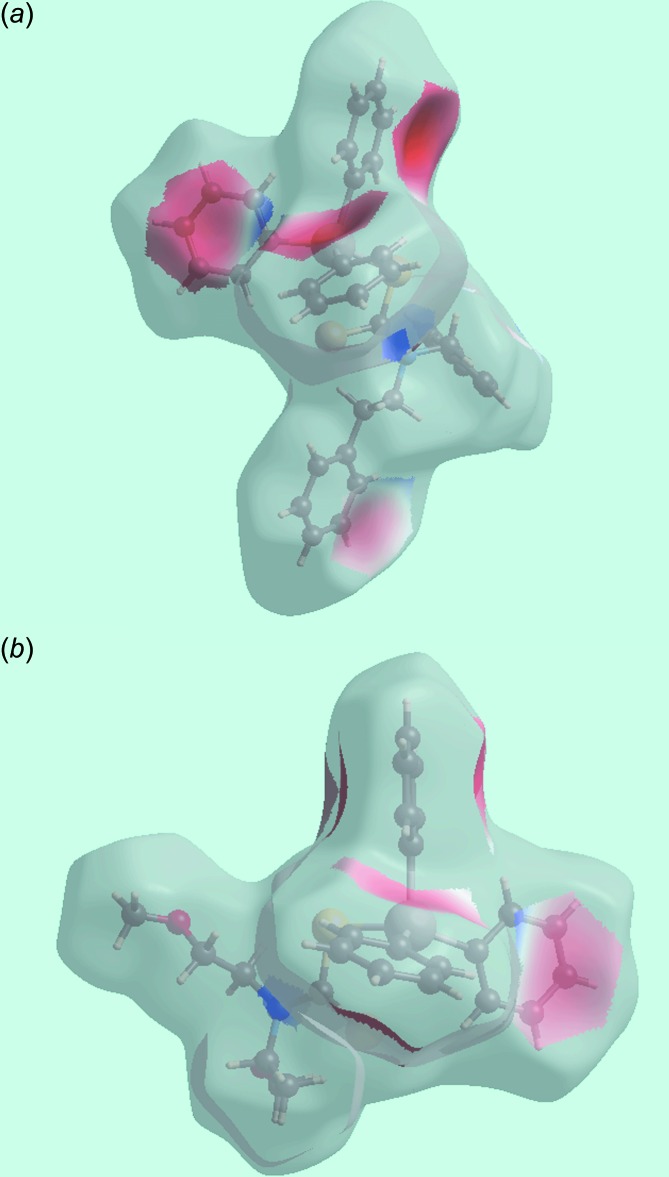
Views of Hirshfeld surfaces mapped over electrostatic potential corresponding to C⋯H contacts (the red spots located near the phenyl rings indicate their contribution as π-bond donors in the C—H⋯π inter­actions) for: (*a*) (I) and (*b*) (II).

**Figure 7:**
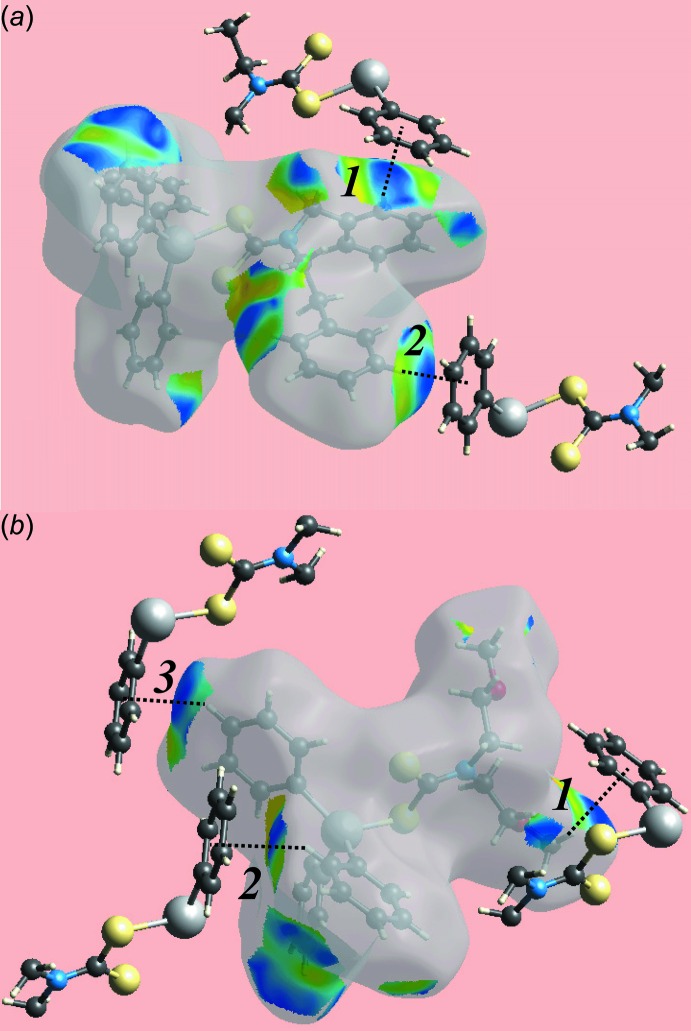
Views of Hirshfeld surfaces mapped over shape-index (*a*) for (I) and (*b*) for (II). The different C—H⋯π contacts are labelled and indicated as dashed lines.

The different shapes of Hirshfeld surfaces for mol­ecules (I) and (II) arise from the asymmetric geometries resulting from the different di­thio­carbamate-bound functional groups, *i.e*. phenyl and meth­oxy groups in (I) and (II), respectively, Fig. 5 ▸. The images of the Hirshfeld surface mapped over electrostatic potential for (I) and (II) display dark-red and dark-blue regions, assigned to negative and positive potentials, respectively, and are localized near their respective functional groups. The absence of conventional hydrogen bonds in the crystals of (I) and (II) is consistent with the non-appearance of characteristic red-spots in the Hirshfeld surface mapped over *d*_norm_ (not shown). The curvature of the Hirshfeld surfaces around the phenyl rings participating as acceptors in the C—H⋯π contacts determine the strength of these inter­actions in the crystal packing. In the structure of (I), the surfaces around the Sn-bound phenyl (C17–C22) and (C23–C28) rings are more concave than the equivalent rings participating in C—H⋯π inter­actions in (II), indicating their greater influence upon packing, as seen in the shorter H⋯ring centroid separations, Tables 2 ▸ and 3 ▸. This observation is also apparent from the Hirshfeld surfaces mapped over electrostatic potential corresponding to C⋯H contacts for (I) and (II), both showing red spots in the images of Fig. 6 ▸ correlating with their functioning as π-bond acceptors. The concave appearance of the Hirshfeld surface mapped over electrostatic potential around the Sn-bound phenyl ring (C14–C19) in the structure of (II) is indicative of its participation in two C—H⋯ π inter­actions, *i.e*. with the H13 and H23 hydrogen atoms. The other C—H⋯π contact involves methyl-H7*C* atom as the donor and phenyl (C8–C13) ring as the acceptor. The shape-indexed Hirshfeld surfaces highlighting the C—H⋯π contacts are shown in Fig. 7 ▸.

**Figure 8:**
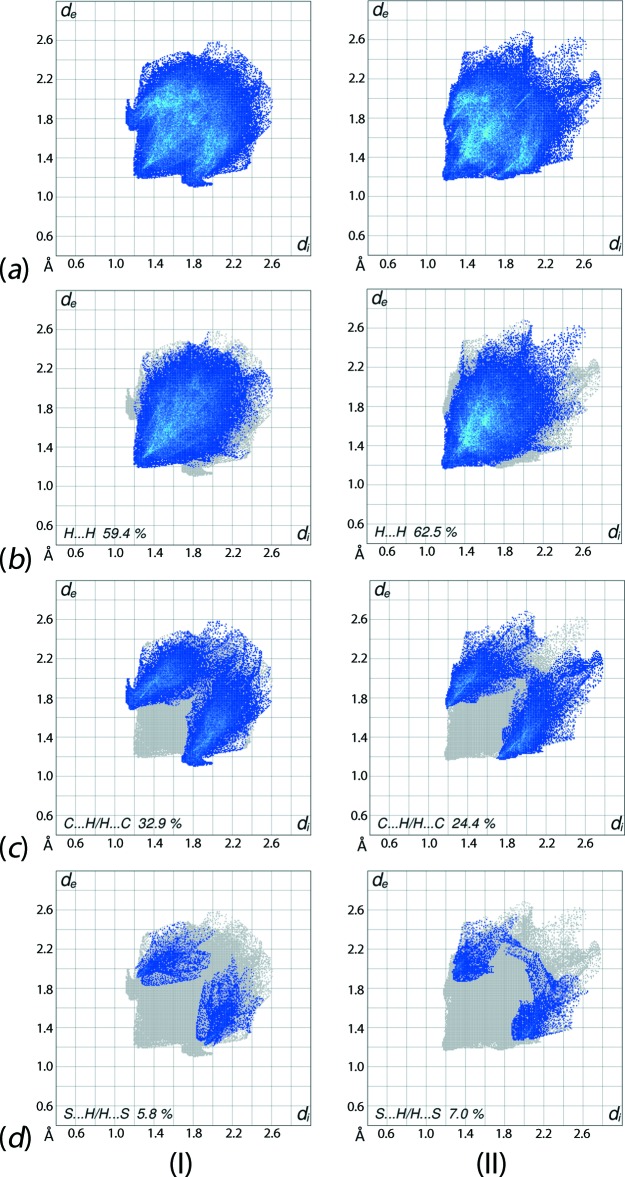
Comparison between (I) and (II) of the (*a*) full two-dimensional fingerprint plots, and the plots delineated into (*b*) H⋯H, (*c*) C⋯H/H⋯C and (*d*) S⋯H/H⋯S contacts.

The overall two-dimensional fingerprint plots for (I) and (II) and those delineated into H⋯H, C⋯H/H⋯C and S⋯H/H⋯S contacts (McKinnon *et al.*, 2007 ▸) are illustrated in Fig. 8 ▸ *a*–*d*, respectively; their relative contributions are summarized in Table 4 ▸. Although the distribution of points in the overall plots of (I) and (II) have almost same (*d*_e_, *d*_i_) ranges, *i.e*. between 1.2 and 2.6 Å, the densities and the areas of their distributions are different. It is evident from the data in Table 4 ▸ and the fingerprint plot delineated into H⋯H contacts in Fig. 8 ▸ *b* that these contacts make the most significant contribution to the Hirshfeld surfaces of both (I) and (II). In the fingerprint plot of (I) delineated into H⋯H contacts (Fig. 8 ▸ *b*), all the points are situated at the (*d*_e_, *d*_i_) distances greater than or equal to their van der Waals separations *i.e*. 2 x 1.2 Å, hence there is no propensity to form such inter­molecular contacts. The peak at (*d*_e_, *d*_i_) distances slightly less than van der Waals separations in the fingerprint plot for (II) is due to a short inter­atomic H⋯H contact between symmetry-related meth­oxy- and di­thio­carbamate hydrogen atoms [H7*A*⋯H5*A* ^i^ = 2.36 Å; symmetry code: (i) −*x*, 2 − *y*, 1 − *z*]. In the fingerprint plot delineated into C⋯H/H⋯C contacts for (I), Fig. 8 ▸ *c*, the 32.9% contribution to the Hirshfeld surface and the symmetrical distribution of points showing bending of the pattern at (*d*_e_ + *d*_i_)_min_ ∼2.8 Å is the result of short inter­atomic C⋯H/H⋯C contacts [C1⋯H32^ii^ = 2.85 and C14⋯H27^iii^ = 2.84 Å; symmetry codes: (ii) 1 + *x*, *y*, *z*; (iii) 1 − *x*, 2 − *y*, −*z*]. In the structure of (II), a comparatively reduced contribution from these contacts to the surface is made, *i.e*. 24.4%, an observation ascribed to the presence of only C—H⋯π contacts in the mol­ecular packing, with no other short inter-atomic contacts. The negligible contribution from C⋯C contacts to the Hirshfeld surfaces indicate that despite the presence of three Sn-bound phenyl rings in the structures of both (I) and (II), and the presence of other two phenyl rings bound to the di­thio­carbamate ligand in (I), the structures show no significant π–π stacking. In the structure of (II), the presence of oxygen atoms does not have any significant influence on its mol­ecular packing although there is 4.7% contribution from O⋯H/H⋯O contacts to the Hirshfeld surface. The fingerprint plots delineated into S⋯H/H⋯S contacts for both the mol­ecules (I) and (II), Fig. 8 ▸ *d*, show that crowded geometries around the tin atoms prevent the sulfur atoms from forming such inter­molecular contacts although these contacts have significant contributions to their respective Hirshfeld surfaces, Table 4 ▸, as well as nearly symmetrical distributions of points in their plots. This observation was also noted in an earlier study describing related organotin di­thio­carbamate structures (Mohamad *et al.*, 2016 ▸).

## Database survey   

According to a search of the Cambridge Structural Database (CSD; Groom *et al.*, 2016 ▸), the di­thio­carbamate ligands featuring in the present study have comparatively rare *R*/*R*′ substituents. For example, the ^−^S_2_CN(Ben)CH_2_CH_2_Ph anion in (I) has only one precedent, namely in Pb[S_2_CN(Ben)CH_2_CH_2_Ph]_2_ (Sathiyaraj *et al.*, 2012 ▸). There are eight examples of the ^−^S_2_CN(CH_2_CH_2_OMe)_2_ anion, as in (II), being the focus of two recent systematic studies (Hogarth *et al.*, 2009 ▸; Naeem *et al.*, 2010 ▸).

Reflecting the inter­est in organotin di­thio­carbamates, there are approximately 40 examples of tri­phenyl­tin di­thio­carbamate structures in the CSD, all of which present the same basic structural motif as described herein for (I) and (II). The prototype compound, Ph_3_Sn(S_2_CNEt_2_) features the shortest Sn—S bond length of the series at 2.429 (3) Å (Hook *et al.*, 1994 ▸). The most asymmetric mode of coordination of a di­thio­carbamate ligand, *i.e*. with Δ(Sn—S) of 0.74 Å, is found in the structure of Ph_3_Sn(4-nitro­phenyl­piperazine-1-di­thio­carbamate) (Rehman *et al.*, 2009 ▸). On the other hand, the most symmetric mode of coordination is found in the structure of Ph_3_Sn(4-meth­oxy­phenyl­piperazine-1-di­thio­carbamate), having Δ(Sn—S) of 0.42 Å (Zia-ur-Rehman *et al.*, 2011 ▸), *i.e*. the same value as found in the structure of (I) reported herein.

## Synthesis and crystallization   

Synthesis of (I): *N*-Benzyl-2-phenyl­ethyl­amine (2 mmol) dissolved in ethanol (10 ml) was stirred for 30 min in an ice-bath. 25% ammonia (1–2 ml) was added to generate a basic solution. After that, a cold ethano­lic solution of carbon di­sulfide (2 mmol) was added to the solution followed by stirring for about 2 h. Then, tri­phenyl­tin(IV) chloride (2 mmol) dissolved in ethanol (30 ml) was added drop wise into the solution followed by further stirring for 2 h. The precipitate that formed was filtered off and washed with cold ethanol a few times to remove impurities. Finally, the precipitate was dried in a desiccator. Recrystallization was achieved by dissolv­ing the compound in a chloro­form and ethanol mixture (1:1 *v*/*v*): this solution was allowed to slowly evaporate at room temperature yielding colourless slabs of (I). M.p.: 419–421 K. Yield: 85%. Analysis: found C, 64.5; H, 5.3; N, 2.3; S, 9.9. C_34_H_31_NS_2_Sn requires: C, 64.2; H, 4.9; N, 2.2; S, 10.1. IR (cm^−1^): 1476 ν(C—N), 1021 ν(C—S), 502 ν(Sn—C), 448 ν(Sn—S). ^1^H NMR (CDCl_3_): 7.44–7.86 (15H, Sn—Ph), 7.16–7.39 (10H, C-Ph), 5.03 (2H, CH_2_Ben), 3.96 (2H, NC*H*_2_CH_2_), 3.04 (2H, NCH_2_C*H*_2_). ^13^C{^1^H} NMR (CDCl_3_): δ 197.8 (S_2_C), 126.7–142.3 (Ar), 59.8 (CH_2_Ben), 56.4 (N*C*H_2_CH_2_), 32.8 (NCH_2_ *C*H_2_). ^119^Sn{^1^H} NMR (CDCl_3_): −180.2.

Compound (II) was prepared in essentially the same manner as for (I) but using bis­(2-meth­oxy­eth­yl)amine (5 mmol) in place of *N*-benzyl-2-phenyl­ethyl­amine. Recrystallization was from chloro­form solution to yield colourless slabs. M.p.: 366–367 K. Yield: 89%. Analysis: found C, 54.4; H, 4.4; N, 2.9; S, 12.1. C_25_H_29_NO_2_S_2_ Sn requires: C, 53.8; H, 5.2; N, 2.5; S, 11.5. IR (cm^−1^): 1470 ν(C—N), 994 ν(C—S), 559 ν(Sn—C), 425 ν(Sn—S). ^1^H NMR (CDCl_3_): 7.40–7.74 (15H, Sn—Ph), 4.13 (2H, OCH_2_), 3.72 (2H, NCH_2_), 3.35 (3H, CH_3_). ^13^C{^1^H} NMR (CDCl_3_): δ 197.3 (S_2_C), 128.6–142.4 (Ar), 70.0 (OCH_2_), 59.0 (NCH_2_), 57.1 (CH_3_). ^119^Sn{^1^H} NMR (CDCl_3_): −185.0.

## Refinement   

Crystal data, data collection and structure refinement details are summarized in Table 5 ▸. Carbon-bound H atoms were placed in calculated positions (C—H = 0.95–0.99 Å) and were included in the refinement in the riding-model approximation, with *U*_iso_(H) set to 1.2–1.5*U*_eq_(C). In the refinement of (II), disorder was noted in the C5-chain of the di­thio­carbamate ligand. Specifically, the C6 and O2 atoms were modelled over two positions in the ratio 0.569 (2):0.431 (2). The anisotropic displacement parameters for both components of the C6 and O5 atoms were constrained to be equivalent; further, those for the C6 atoms were restrained to be approximately isotropic. The 1,2 and 1,3 bond lengths of the disordered residual were restrained to be similar to those of the ordered arm of the di­thio­carbamate ligand.

## Supplementary Material

Crystal structure: contains datablock(s) I, II, global. DOI: 10.1107/S2056989016014985/hb7618sup1.cif

Structure factors: contains datablock(s) I. DOI: 10.1107/S2056989016014985/hb7618Isup2.hkl

Structure factors: contains datablock(s) II. DOI: 10.1107/S2056989016014985/hb7618IIsup3.hkl

CCDC references: 1505733, 1505732

Additional supporting information:  crystallographic information; 3D view; checkCIF report

## Figures and Tables

**Table 1:** Geometric data (Å, °) for (I) and (II)

Parameter	(I)	(II)
Sn—S1	2.4886 (4)	2.4612 (4)
Sn—S2	2.9120 (3)	3.0992 (4)
Sn—C17	2.1696 (13)	–
Sn—C23	2.1309 (13)	–
Sn—C29	2.1469 (13)	–
Sn—C8	–	2.1312 (14)
Sn—C14	–	2.1608 (14)
Sn—C20	–	2.1357 (15)
C1—S1	1.7532 (13)	1.7629 (14)
C1—S2	1.6902 (13)	1.6781 (14)
S1—Sn—S2	65.919 (10)	63.534 (11)
S2—Sn—C17	158.55 (4)	–
S2—Sn—C14	–	154.45 (4)

**Table 2:** Hydrogen-bond geometry (Å, °) for (I) *Cg*1 and *Cg*2 are the centroids of the C17–C22 and C23–C28 rings, respectively.

*D*—H⋯*A*	*D*—H	H⋯*A*	*D*⋯*A*	*D*—H⋯*A*
C4—H4⋯*Cg*1^i^	0.95	2.63	3.4732 (17)	148
C13—H13⋯*Cg*2^ii^	0.95	2.62	3.5227 (17)	159

Symmetry codes: (i) 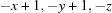; (ii) 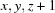.

**Table 3:** Hydrogen-bond geometry (Å, °) for (II) *Cg*1 and *Cg*2 are the centroids of the C8–C13 and C14–C19 rings, respectively.

*D*—H⋯*A*	*D*—H	H⋯*A*	*D*⋯*A*	*D*—H⋯*A*
C7—H7*C*⋯*Cg*1^i^	0.98	2.94	3.821 (3)	151
C13—H13⋯*Cg*2^ii^	0.95	2.98	3.7979 (18)	145
C23—H23⋯*Cg*2^iii^	0.95	2.97	3.707 (2)	136

Symmetry codes: (i) 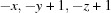; (ii) 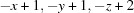; (iii) 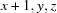.

**Table 4:** Percentage contribution to inter­atomic contacts from the Hirshfeld surface for (I) and (II)

Contact	(I)	(II)
H⋯H	59.4	62.5
C⋯H/H⋯C	32.9	24.4
O⋯H/H⋯O	–	4.7
S⋯H/H⋯S	5.8	7.0
C⋯S/S⋯C	0.4	0.0
N⋯H/H⋯N	0.5	0.4
C⋯C	0.9	0.0
S⋯S	0.0	0.4
C⋯O/O⋯C	0.1	0.1
O⋯O	–	0.5

**Table 5:** Experimental details

	(I)	(II)
Crystal data		
Chemical formula	[Sn(C_6_H_5_)_3_(C_16_H_16_NS_2_)]	[Sn(C_6_H_5_)_3_(C_7_H_14_NO_2_S_2_)]
*M*_r_	636.41	558.35
Crystal system, space group	Triclinic, *P* 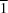	Triclinic, *P* 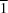
Temperature (K)	139	147
*a*, *b*, *c* (Å)	9.5856 (2), 11.6140 (2), 13.6795 (3)	9.6703 (2), 9.8015 (2), 13.8515 (3)
α, β, γ (°)	78.043 (2), 77.868 (2), 82.358 (2)	95.092 (2), 99.467 (2), 105.841 (2)
*V* (Å^3^)	1450.20 (5)	1233.41 (5)
*Z*	2	2
Radiation type	Mo *K*α	Mo *K*α
μ (mm^−1^)	1.05	1.23
Crystal size (mm)	0.50 × 0.30 × 0.20	0.50 × 0.50 × 0.20

Data collection		
Diffractometer	Agilent Technologies SuperNova Dual diffractometer with an Atlas detector	Agilent Technologies SuperNova Dual diffractometer with an Atlas detector
Absorption correction	Multi-scan (*CrysAlis PRO*; Agilent, 2015 ▸)	Multi-scan (*CrysAlis PRO*; Agilent, 2015 ▸)
*T*_min_, *T*_max_	0.804, 1.000	0.722, 1.000
No. of measured, independent and observed [*I* > 2σ(*I*)] reflections	41508, 9103, 8428	35286, 7773, 7157
*R*_int_	0.038	0.035
(sin θ/λ)_max_ (Å^−1^)	0.741	0.740

Refinement		
*R*[*F* ^2^ > 2σ(*F* ^2^)], *wR*(*F* ^2^), *S*	0.022, 0.055, 1.00	0.023, 0.056, 1.03
No. of reflections	9103	7773
No. of parameters	343	290
No. of restraints	0	18
H-atom treatment	H-atom parameters constrained	H-atom parameters constrained
Δρ_max_, Δρ_min_ (e Å^−3^)	0.53, −0.61	0.55, −0.61

Computer programs: *CrysAlis PRO* (Agilent, 2015 ▸), *SHELXS97* (Sheldrick, 2008 ▸), *SHELXL2014* (Sheldrick, 2015 ▸), *ORTEP-3 for Windows* (Farrugia, 2012 ▸), *DIAMOND* (Brandenburg, 2006 ▸) and *publCIF* (Westrip, 2010 ▸).

## supplementary crystallographic information

### (I) [N-Benzyl-N-(2-phenylethyl)dithiocarbamato-κ2S,S']triphenyltin(IV) Crystal data

**Table:**

[Sn(C_6_H_5_)_3_(C_16_H_16_NS_2_)]	*Z* = 2
*M**_r_* = 636.41	*F*(000) = 648
Triclinic, *P*1	*D*_x_ = 1.457 Mg m^−^^3^
*a* = 9.5856 (2) Å	Mo *K*α radiation, λ = 0.71073 Å
*b* = 11.6140 (2) Å	Cell parameters from 26203 reflections
*c* = 13.6795 (3) Å	θ = 4.1–31.4°
α = 78.043 (2)°	µ = 1.05 mm^−^^1^
β = 77.868 (2)°	*T* = 139 K
γ = 82.358 (2)°	Slab, colourless
*V* = 1450.20 (5) Å^3^	0.50 × 0.30 × 0.20 mm

### (I) [N-Benzyl-N-(2-phenylethyl)dithiocarbamato-κ2S,S']triphenyltin(IV) Data collection

**Table:**

Agilent Technologies SuperNova Dual diffractometer with an Atlas detector	9103 independent reflections
Radiation source: SuperNova (Mo) X-ray Source	8428 reflections with *I* > 2σ(*I*)
Mirror monochromator	*R*_int_ = 0.038
Detector resolution: 10.4041 pixels mm^-1^	θ_max_ = 31.8°, θ_min_ = 3.3°
ω scan	*h* = −13→14
Absorption correction: multi-scan (CrysAlis PRO; Agilent, 2015)	*k* = −17→17
*T*_min_ = 0.804, *T*_max_ = 1.000	*l* = −19→19
41508 measured reflections	

### (I) [N-Benzyl-N-(2-phenylethyl)dithiocarbamato-κ2S,S']triphenyltin(IV) Refinement

**Table:**

Refinement on *F*^2^	0 restraints
Least-squares matrix: full	Hydrogen site location: inferred from neighbouring sites
*R*[*F*^2^ > 2σ(*F*^2^)] = 0.022	H-atom parameters constrained
*wR*(*F*^2^) = 0.055	*w* = 1/[σ^2^(*F*_o_^2^) + (0.0236*P*)^2^ + 0.5974*P*] where *P* = (*F*_o_^2^ + 2*F*_c_^2^)/3
*S* = 1.00	(Δ/σ)_max_ = 0.003
9103 reflections	Δρ_max_ = 0.53 e Å^−^^3^
343 parameters	Δρ_min_ = −0.61 e Å^−^^3^

### (I) [N-Benzyl-N-(2-phenylethyl)dithiocarbamato-κ2S,S']triphenyltin(IV) Special details

**Table:**

Geometry. All esds (except the esd in the dihedral angle between two l.s. planes) are estimated using the full covariance matrix. The cell esds are taken into account individually in the estimation of esds in distances, angles and torsion angles; correlations between esds in cell parameters are only used when they are defined by crystal symmetry. An approximate (isotropic) treatment of cell esds is used for estimating esds involving l.s. planes.

### (I) [N-Benzyl-N-(2-phenylethyl)dithiocarbamato-κ2S,S']triphenyltin(IV) Fractional atomic coordinates and isotropic or equivalent isotropic displacement parameters (Å^2^)

**Table:**

	*x*	*y*	*z*	*U*_iso_*/*U*_eq_	
Sn	0.11097 (2)	0.73083 (2)	−0.11080 (2)	0.01757 (3)	
S1	0.26398 (4)	0.60268 (3)	0.00262 (3)	0.02307 (7)	
S2	0.14838 (4)	0.83511 (3)	0.05703 (3)	0.02241 (7)	
N1	0.34474 (13)	0.69202 (11)	0.14450 (9)	0.0226 (2)	
C1	0.25975 (14)	0.71037 (11)	0.07630 (10)	0.0195 (2)	
C2	0.45063 (16)	0.58852 (14)	0.15813 (11)	0.0276 (3)	
H2A	0.5464	0.6161	0.1504	0.033*	
H2B	0.4549	0.5425	0.1040	0.033*	
C3	0.41603 (15)	0.50885 (13)	0.26046 (11)	0.0242 (3)	
C4	0.51461 (16)	0.48312 (14)	0.32492 (12)	0.0299 (3)	
H4	0.6025	0.5186	0.3057	0.036*	
C5	0.48559 (19)	0.40590 (17)	0.41719 (13)	0.0383 (4)	
H5	0.5538	0.3884	0.4607	0.046*	
C6	0.3580 (2)	0.35456 (16)	0.44587 (14)	0.0402 (4)	
H6	0.3389	0.3007	0.5086	0.048*	
C7	0.25767 (19)	0.38180 (15)	0.38302 (15)	0.0390 (4)	
H7	0.1689	0.3477	0.4033	0.047*	
C8	0.28613 (17)	0.45813 (14)	0.29129 (13)	0.0317 (3)	
H8	0.2167	0.4764	0.2486	0.038*	
C9	0.34155 (15)	0.78002 (13)	0.20882 (11)	0.0252 (3)	
H9A	0.3358	0.8602	0.1667	0.030*	
H9B	0.4319	0.7680	0.2353	0.030*	
C10	0.21466 (18)	0.77186 (14)	0.29854 (11)	0.0293 (3)	
H10A	0.1252	0.7726	0.2731	0.035*	
H10B	0.2276	0.6961	0.3461	0.035*	
C11	0.20107 (15)	0.87303 (13)	0.35476 (10)	0.0226 (3)	
C12	0.22314 (16)	0.85318 (14)	0.45412 (11)	0.0268 (3)	
H12	0.2495	0.7751	0.4866	0.032*	
C13	0.20723 (17)	0.94591 (15)	0.50684 (11)	0.0298 (3)	
H13	0.2219	0.9310	0.5751	0.036*	
C14	0.17019 (17)	1.05948 (14)	0.45984 (12)	0.0309 (3)	
H14	0.1583	1.1229	0.4958	0.037*	
C15	0.15027 (17)	1.08086 (14)	0.35992 (13)	0.0306 (3)	
H15	0.1260	1.1593	0.3272	0.037*	
C16	0.16559 (16)	0.98869 (14)	0.30788 (11)	0.0273 (3)	
H16	0.1518	1.0042	0.2394	0.033*	
C17	0.11990 (14)	0.59670 (12)	−0.20181 (10)	0.0202 (2)	
C18	0.12215 (15)	0.62884 (12)	−0.30614 (11)	0.0226 (3)	
H18	0.1261	0.7095	−0.3379	0.027*	
C19	0.11864 (16)	0.54453 (14)	−0.36467 (12)	0.0280 (3)	
H19	0.1215	0.5677	−0.4358	0.034*	
C20	0.11097 (17)	0.42673 (14)	−0.31871 (13)	0.0318 (3)	
H20	0.1072	0.3693	−0.3582	0.038*	
C21	0.10880 (17)	0.39296 (13)	−0.21536 (13)	0.0317 (3)	
H21	0.1038	0.3122	−0.1840	0.038*	
C22	0.11387 (15)	0.47709 (12)	−0.15718 (11)	0.0253 (3)	
H22	0.1132	0.4530	−0.0864	0.030*	
C23	0.23372 (14)	0.86620 (12)	−0.20545 (10)	0.0196 (2)	
C24	0.18559 (18)	0.98566 (13)	−0.21328 (12)	0.0290 (3)	
H24	0.0933	1.0088	−0.1776	0.035*	
C25	0.2715 (2)	1.07114 (15)	−0.27286 (14)	0.0412 (4)	
H25	0.2374	1.1524	−0.2779	0.049*	
C26	0.4055 (2)	1.03887 (18)	−0.32465 (14)	0.0444 (4)	
H26	0.4642	1.0977	−0.3648	0.053*	
C27	0.45457 (18)	0.92100 (19)	−0.31816 (15)	0.0434 (4)	
H27	0.5470	0.8986	−0.3541	0.052*	
C28	0.36913 (15)	0.83497 (15)	−0.25919 (12)	0.0306 (3)	
H28	0.4034	0.7539	−0.2554	0.037*	
C29	−0.11619 (14)	0.77083 (11)	−0.06779 (10)	0.0194 (2)	
C30	−0.18103 (15)	0.85380 (12)	−0.00804 (12)	0.0256 (3)	
H30	−0.1239	0.8910	0.0229	0.031*	
C31	−0.32861 (16)	0.88286 (14)	0.00684 (13)	0.0320 (3)	
H31	−0.3716	0.9393	0.0482	0.038*	
C32	−0.41312 (16)	0.82989 (14)	−0.03838 (13)	0.0318 (3)	
H32	−0.5136	0.8511	−0.0293	0.038*	
C33	−0.35052 (16)	0.74601 (14)	−0.09672 (13)	0.0318 (3)	
H33	−0.4082	0.7085	−0.1270	0.038*	
C34	−0.20313 (15)	0.71655 (13)	−0.11116 (12)	0.0256 (3)	
H34	−0.1610	0.6586	−0.1511	0.031*	

### (I) [N-Benzyl-N-(2-phenylethyl)dithiocarbamato-κ2S,S']triphenyltin(IV) Atomic displacement parameters (Å^2^)

**Table:**

	*U*^11^	*U*^22^	*U*^33^	*U*^12^	*U*^13^	*U*^23^
Sn	0.01871 (5)	0.01729 (5)	0.01588 (4)	−0.00169 (3)	−0.00165 (3)	−0.00279 (3)
S1	0.02930 (17)	0.01917 (15)	0.02176 (15)	0.00255 (12)	−0.00751 (13)	−0.00641 (12)
S2	0.02570 (16)	0.02023 (15)	0.02146 (15)	0.00247 (12)	−0.00597 (13)	−0.00562 (12)
N1	0.0248 (5)	0.0254 (6)	0.0181 (5)	0.0031 (4)	−0.0055 (4)	−0.0067 (4)
C1	0.0213 (6)	0.0199 (6)	0.0164 (6)	−0.0022 (5)	−0.0008 (5)	−0.0034 (5)
C2	0.0258 (7)	0.0334 (8)	0.0221 (7)	0.0085 (6)	−0.0061 (5)	−0.0070 (6)
C3	0.0267 (7)	0.0236 (6)	0.0234 (6)	0.0049 (5)	−0.0075 (5)	−0.0085 (5)
C4	0.0242 (7)	0.0363 (8)	0.0281 (7)	0.0037 (6)	−0.0081 (6)	−0.0044 (6)
C5	0.0348 (8)	0.0473 (10)	0.0287 (8)	0.0094 (7)	−0.0119 (7)	−0.0003 (7)
C6	0.0443 (9)	0.0324 (8)	0.0345 (9)	0.0058 (7)	−0.0027 (7)	0.0039 (7)
C7	0.0360 (8)	0.0285 (8)	0.0507 (11)	−0.0051 (6)	−0.0076 (8)	−0.0026 (7)
C8	0.0331 (8)	0.0281 (7)	0.0384 (9)	−0.0019 (6)	−0.0158 (7)	−0.0078 (7)
C9	0.0276 (7)	0.0302 (7)	0.0204 (6)	−0.0024 (5)	−0.0055 (5)	−0.0095 (6)
C10	0.0392 (8)	0.0292 (7)	0.0190 (6)	−0.0071 (6)	0.0008 (6)	−0.0075 (6)
C11	0.0238 (6)	0.0262 (7)	0.0173 (6)	−0.0025 (5)	−0.0016 (5)	−0.0050 (5)
C12	0.0318 (7)	0.0289 (7)	0.0173 (6)	−0.0015 (6)	−0.0034 (5)	−0.0012 (5)
C13	0.0329 (8)	0.0406 (8)	0.0177 (6)	−0.0091 (6)	−0.0030 (6)	−0.0078 (6)
C14	0.0316 (7)	0.0323 (8)	0.0296 (8)	−0.0094 (6)	0.0037 (6)	−0.0132 (6)
C15	0.0322 (7)	0.0253 (7)	0.0315 (8)	−0.0013 (6)	−0.0025 (6)	−0.0032 (6)
C16	0.0313 (7)	0.0309 (7)	0.0191 (6)	−0.0008 (6)	−0.0079 (5)	−0.0014 (5)
C17	0.0188 (6)	0.0201 (6)	0.0204 (6)	−0.0017 (4)	−0.0007 (5)	−0.0043 (5)
C18	0.0247 (6)	0.0219 (6)	0.0214 (6)	−0.0005 (5)	−0.0059 (5)	−0.0043 (5)
C19	0.0280 (7)	0.0350 (8)	0.0239 (7)	−0.0028 (6)	−0.0057 (6)	−0.0111 (6)
C20	0.0306 (7)	0.0329 (8)	0.0358 (8)	−0.0073 (6)	−0.0007 (6)	−0.0184 (7)
C21	0.0368 (8)	0.0213 (7)	0.0355 (8)	−0.0087 (6)	0.0034 (7)	−0.0082 (6)
C22	0.0291 (7)	0.0224 (6)	0.0219 (6)	−0.0052 (5)	0.0018 (5)	−0.0037 (5)
C23	0.0213 (6)	0.0220 (6)	0.0163 (6)	−0.0052 (5)	−0.0045 (5)	−0.0026 (5)
C24	0.0401 (8)	0.0226 (7)	0.0235 (7)	−0.0042 (6)	−0.0003 (6)	−0.0072 (6)
C25	0.0680 (12)	0.0241 (7)	0.0330 (9)	−0.0168 (8)	−0.0050 (8)	−0.0051 (7)
C26	0.0520 (11)	0.0491 (11)	0.0348 (9)	−0.0343 (9)	−0.0072 (8)	0.0039 (8)
C27	0.0230 (7)	0.0605 (12)	0.0408 (10)	−0.0135 (7)	−0.0005 (7)	0.0044 (9)
C28	0.0207 (6)	0.0349 (8)	0.0315 (8)	−0.0002 (6)	−0.0023 (6)	0.0003 (6)
C29	0.0196 (6)	0.0180 (6)	0.0180 (6)	−0.0014 (4)	−0.0014 (5)	0.0003 (5)
C30	0.0249 (6)	0.0223 (6)	0.0288 (7)	0.0006 (5)	−0.0033 (5)	−0.0065 (6)
C31	0.0257 (7)	0.0266 (7)	0.0391 (9)	0.0043 (6)	0.0012 (6)	−0.0073 (6)
C32	0.0194 (6)	0.0285 (7)	0.0411 (9)	0.0002 (5)	−0.0004 (6)	0.0013 (6)
C33	0.0235 (7)	0.0336 (8)	0.0392 (9)	−0.0053 (6)	−0.0079 (6)	−0.0050 (7)
C34	0.0236 (6)	0.0255 (7)	0.0276 (7)	−0.0026 (5)	−0.0039 (5)	−0.0055 (6)

### (I) [N-Benzyl-N-(2-phenylethyl)dithiocarbamato-κ2S,S']triphenyltin(IV) Geometric parameters (Å, º)

**Table:**

Sn—C23	2.1309 (13)	C14—H14	0.9500
Sn—C29	2.1469 (13)	C15—C16	1.380 (2)
Sn—C17	2.1696 (13)	C15—H15	0.9500
Sn—S1	2.4886 (4)	C16—H16	0.9500
Sn—S2	2.9120 (3)	C17—C18	1.3947 (19)
S1—C1	1.7532 (13)	C17—C22	1.4003 (19)
S2—C1	1.6902 (13)	C18—C19	1.3949 (19)
N1—C1	1.3305 (18)	C18—H18	0.9500
N1—C9	1.4739 (17)	C19—C20	1.387 (2)
N1—C2	1.4739 (17)	C19—H19	0.9500
C2—C3	1.508 (2)	C20—C21	1.383 (2)
C2—H2A	0.9900	C20—H20	0.9500
C2—H2B	0.9900	C21—C22	1.393 (2)
C3—C4	1.388 (2)	C21—H21	0.9500
C3—C8	1.395 (2)	C22—H22	0.9500
C4—C5	1.387 (2)	C23—C24	1.3922 (19)
C4—H4	0.9500	C23—C28	1.3932 (19)
C5—C6	1.379 (3)	C24—C25	1.388 (2)
C5—H5	0.9500	C24—H24	0.9500
C6—C7	1.385 (3)	C25—C26	1.376 (3)
C6—H6	0.9500	C25—H25	0.9500
C7—C8	1.376 (2)	C26—C27	1.378 (3)
C7—H7	0.9500	C26—H26	0.9500
C8—H8	0.9500	C27—C28	1.388 (2)
C9—C10	1.533 (2)	C27—H27	0.9500
C9—H9A	0.9900	C28—H28	0.9500
C9—H9B	0.9900	C29—C30	1.3915 (18)
C10—C11	1.5102 (19)	C29—C34	1.3952 (19)
C10—H10A	0.9900	C30—C31	1.392 (2)
C10—H10B	0.9900	C30—H30	0.9500
C11—C12	1.3869 (19)	C31—C32	1.385 (2)
C11—C16	1.394 (2)	C31—H31	0.9500
C12—C13	1.391 (2)	C32—C33	1.383 (2)
C12—H12	0.9500	C32—H32	0.9500
C13—C14	1.378 (2)	C33—C34	1.391 (2)
C13—H13	0.9500	C33—H33	0.9500
C14—C15	1.387 (2)	C34—H34	0.9500
			
C23—Sn—C29	118.33 (5)	C13—C14—C15	119.82 (14)
C23—Sn—C17	106.09 (5)	C13—C14—H14	120.1
C29—Sn—C17	101.34 (5)	C15—C14—H14	120.1
C23—Sn—S1	108.24 (4)	C16—C15—C14	120.25 (15)
C29—Sn—S1	124.31 (4)	C16—C15—H15	119.9
C17—Sn—S1	92.98 (4)	C14—C15—H15	119.9
C23—Sn—S2	85.28 (3)	C15—C16—C11	120.67 (14)
C29—Sn—S2	88.48 (4)	C15—C16—H16	119.7
C17—Sn—S2	158.55 (4)	C11—C16—H16	119.7
S1—Sn—S2	65.919 (10)	C18—C17—C22	118.10 (12)
C1—S1—Sn	93.73 (5)	C18—C17—Sn	120.38 (10)
C1—S2—Sn	81.22 (5)	C22—C17—Sn	121.39 (10)
C1—N1—C9	120.43 (11)	C17—C18—C19	121.14 (13)
C1—N1—C2	123.71 (11)	C17—C18—H18	119.4
C9—N1—C2	115.81 (11)	C19—C18—H18	119.4
N1—C1—S2	122.25 (10)	C20—C19—C18	119.81 (14)
N1—C1—S1	119.24 (10)	C20—C19—H19	120.1
S2—C1—S1	118.51 (8)	C18—C19—H19	120.1
N1—C2—C3	112.93 (11)	C21—C20—C19	119.96 (14)
N1—C2—H2A	109.0	C21—C20—H20	120.0
C3—C2—H2A	109.0	C19—C20—H20	120.0
N1—C2—H2B	109.0	C20—C21—C22	120.16 (14)
C3—C2—H2B	109.0	C20—C21—H21	119.9
H2A—C2—H2B	107.8	C22—C21—H21	119.9
C4—C3—C8	118.84 (15)	C21—C22—C17	120.82 (14)
C4—C3—C2	120.27 (14)	C21—C22—H22	119.6
C8—C3—C2	120.88 (13)	C17—C22—H22	119.6
C5—C4—C3	120.38 (15)	C24—C23—C28	118.41 (13)
C5—C4—H4	119.8	C24—C23—Sn	122.40 (10)
C3—C4—H4	119.8	C28—C23—Sn	119.16 (10)
C6—C5—C4	120.19 (16)	C23—C24—C25	120.47 (15)
C6—C5—H5	119.9	C23—C24—H24	119.8
C4—C5—H5	119.9	C25—C24—H24	119.8
C5—C6—C7	119.79 (17)	C26—C25—C24	120.40 (16)
C5—C6—H6	120.1	C26—C25—H25	119.8
C7—C6—H6	120.1	C24—C25—H25	119.8
C8—C7—C6	120.22 (17)	C25—C26—C27	119.89 (16)
C8—C7—H7	119.9	C25—C26—H26	120.1
C6—C7—H7	119.9	C27—C26—H26	120.1
C7—C8—C3	120.55 (15)	C28—C27—C26	120.08 (16)
C7—C8—H8	119.7	C28—C27—H27	120.0
C3—C8—H8	119.7	C26—C27—H27	120.0
N1—C9—C10	112.51 (12)	C27—C28—C23	120.74 (15)
N1—C9—H9A	109.1	C27—C28—H28	119.6
C10—C9—H9A	109.1	C23—C28—H28	119.6
N1—C9—H9B	109.1	C30—C29—C34	118.28 (12)
C10—C9—H9B	109.1	C30—C29—Sn	124.91 (10)
H9A—C9—H9B	107.8	C34—C29—Sn	116.54 (9)
C11—C10—C9	111.70 (12)	C31—C30—C29	120.74 (14)
C11—C10—H10A	109.3	C31—C30—H30	119.6
C9—C10—H10A	109.3	C29—C30—H30	119.6
C11—C10—H10B	109.3	C30—C31—C32	120.27 (14)
C9—C10—H10B	109.3	C30—C31—H31	119.9
H10A—C10—H10B	107.9	C32—C31—H31	119.9
C12—C11—C16	118.47 (13)	C33—C32—C31	119.65 (14)
C12—C11—C10	120.88 (13)	C33—C32—H32	120.2
C16—C11—C10	120.65 (13)	C31—C32—H32	120.2
C11—C12—C13	120.94 (14)	C32—C33—C34	120.05 (15)
C11—C12—H12	119.5	C32—C33—H33	120.0
C13—C12—H12	119.5	C34—C33—H33	120.0
C14—C13—C12	119.83 (14)	C33—C34—C29	120.98 (13)
C14—C13—H13	120.1	C33—C34—H34	119.5
C12—C13—H13	120.1	C29—C34—H34	119.5
			
C9—N1—C1—S2	1.80 (18)	C13—C14—C15—C16	0.8 (2)
C2—N1—C1—S2	−175.36 (11)	C14—C15—C16—C11	0.1 (2)
C9—N1—C1—S1	−178.87 (10)	C12—C11—C16—C15	−1.2 (2)
C2—N1—C1—S1	3.97 (19)	C10—C11—C16—C15	178.66 (14)
Sn—S2—C1—N1	172.23 (12)	C22—C17—C18—C19	−0.1 (2)
Sn—S2—C1—S1	−7.10 (7)	Sn—C17—C18—C19	−175.86 (10)
Sn—S1—C1—N1	−171.12 (11)	C17—C18—C19—C20	0.8 (2)
Sn—S1—C1—S2	8.24 (8)	C18—C19—C20—C21	−0.9 (2)
C1—N1—C2—C3	−114.69 (15)	C19—C20—C21—C22	0.2 (2)
C9—N1—C2—C3	68.04 (16)	C20—C21—C22—C17	0.6 (2)
N1—C2—C3—C4	−122.90 (15)	C18—C17—C22—C21	−0.6 (2)
N1—C2—C3—C8	58.25 (18)	Sn—C17—C22—C21	175.12 (11)
C8—C3—C4—C5	1.6 (2)	C28—C23—C24—C25	−0.4 (2)
C2—C3—C4—C5	−177.25 (14)	Sn—C23—C24—C25	177.51 (12)
C3—C4—C5—C6	−0.4 (3)	C23—C24—C25—C26	−0.2 (3)
C4—C5—C6—C7	−1.1 (3)	C24—C25—C26—C27	0.6 (3)
C5—C6—C7—C8	1.2 (3)	C25—C26—C27—C28	−0.2 (3)
C6—C7—C8—C3	0.1 (3)	C26—C27—C28—C23	−0.4 (3)
C4—C3—C8—C7	−1.5 (2)	C24—C23—C28—C27	0.7 (2)
C2—C3—C8—C7	177.40 (14)	Sn—C23—C28—C27	−177.25 (13)
C1—N1—C9—C10	79.44 (17)	C34—C29—C30—C31	−0.8 (2)
C2—N1—C9—C10	−103.19 (15)	Sn—C29—C30—C31	173.03 (12)
N1—C9—C10—C11	−171.97 (12)	C29—C30—C31—C32	−0.4 (2)
C9—C10—C11—C12	−113.86 (16)	C30—C31—C32—C33	1.4 (2)
C9—C10—C11—C16	66.32 (19)	C31—C32—C33—C34	−1.0 (2)
C16—C11—C12—C13	1.4 (2)	C32—C33—C34—C29	−0.2 (2)
C10—C11—C12—C13	−178.42 (14)	C30—C29—C34—C33	1.2 (2)
C11—C12—C13—C14	−0.5 (2)	Sn—C29—C34—C33	−173.21 (12)
C12—C13—C14—C15	−0.6 (2)		

### (I) [N-Benzyl-N-(2-phenylethyl)dithiocarbamato-κ2S,S']triphenyltin(IV) Hydrogen-bond geometry (Å, º)

Cg1 and Cg2 are the centroids of the C17–C22 and C23–C28 rings, respectively.

**Table:**

*D*—H···*A*	*D*—H	H···*A*	*D*···*A*	*D*—H···*A*
C4—H4···*Cg*1^i^	0.95	2.63	3.4732 (17)	148
C13—H13···*Cg*2^ii^	0.95	2.62	3.5227 (17)	159

Symmetry codes: (i) −*x*+1, −*y*+1, −*z*; (ii) *x*, *y*, *z*+1.

### (II) [Bis(2-methoxyethyl)dithiocarbamato-κ2S,S']triphenyltin(IV) Crystal data

**Table:**

[Sn(C_6_H_5_)_3_(C_7_H_14_NO_2_S_2_)]	*Z* = 2
*M**_r_* = 558.35	*F*(000) = 568
Triclinic, *P*1	*D*_x_ = 1.503 Mg m^−^^3^
*a* = 9.6703 (2) Å	Mo *K*α radiation, λ = 0.71073 Å
*b* = 9.8015 (2) Å	Cell parameters from 22178 reflections
*c* = 13.8515 (3) Å	θ = 3.5–31.4°
α = 95.092 (2)°	µ = 1.23 mm^−^^1^
β = 99.467 (2)°	*T* = 147 K
γ = 105.841 (2)°	Slab, colourless
*V* = 1233.41 (5) Å^3^	0.50 × 0.50 × 0.20 mm

### (II) [Bis(2-methoxyethyl)dithiocarbamato-κ2S,S']triphenyltin(IV) Data collection

**Table:**

Agilent Technologies SuperNova Dual diffractometer with an Atlas detector	7773 independent reflections
Radiation source: SuperNova (Mo) X-ray Source	7157 reflections with *I* > 2σ(*I*)
Mirror monochromator	*R*_int_ = 0.035
Detector resolution: 10.4041 pixels mm^-1^	θ_max_ = 31.7°, θ_min_ = 3.4°
ω scan	*h* = −14→14
Absorption correction: multi-scan (CrysAlis PRO; Agilent, 2015)	*k* = −14→14
*T*_min_ = 0.722, *T*_max_ = 1.000	*l* = −20→20
35286 measured reflections	

### (II) [Bis(2-methoxyethyl)dithiocarbamato-κ2S,S']triphenyltin(IV) Refinement

**Table:**

Refinement on *F*^2^	18 restraints
Least-squares matrix: full	Hydrogen site location: inferred from neighbouring sites
*R*[*F*^2^ > 2σ(*F*^2^)] = 0.023	H-atom parameters constrained
*wR*(*F*^2^) = 0.056	*w* = 1/[σ^2^(*F*_o_^2^) + (0.0258*P*)^2^ + 0.4182*P*] where *P* = (*F*_o_^2^ + 2*F*_c_^2^)/3
*S* = 1.03	(Δ/σ)_max_ = 0.001
7773 reflections	Δρ_max_ = 0.55 e Å^−^^3^
290 parameters	Δρ_min_ = −0.61 e Å^−^^3^

### (II) [Bis(2-methoxyethyl)dithiocarbamato-κ2S,S']triphenyltin(IV) Special details

**Table:**

Geometry. All esds (except the esd in the dihedral angle between two l.s. planes) are estimated using the full covariance matrix. The cell esds are taken into account individually in the estimation of esds in distances, angles and torsion angles; correlations between esds in cell parameters are only used when they are defined by crystal symmetry. An approximate (isotropic) treatment of cell esds is used for estimating esds involving l.s. planes.

### (II) [Bis(2-methoxyethyl)dithiocarbamato-κ2S,S']triphenyltin(IV) Fractional atomic coordinates and isotropic or equivalent isotropic displacement parameters (Å^2^)

**Table:**

	*x*	*y*	*z*	*U*_iso_*/*U*_eq_	Occ. (<1)
Sn	0.39512 (2)	0.60246 (2)	0.81652 (2)	0.01772 (3)	
S1	0.25030 (4)	0.72645 (4)	0.71341 (3)	0.02167 (7)	
S2	0.40147 (5)	0.56965 (4)	0.59282 (3)	0.02642 (8)	
O1	0.32503 (13)	0.89169 (13)	0.37430 (9)	0.0305 (3)	
C1	0.28578 (16)	0.66905 (15)	0.59818 (10)	0.0193 (3)	
C2	0.23365 (17)	0.66021 (16)	0.41897 (11)	0.0232 (3)	
H2A	0.1416	0.6499	0.3716	0.028*	
H2B	0.2501	0.5648	0.4179	0.028*	
C3	0.35905 (18)	0.76178 (17)	0.38579 (12)	0.0260 (3)	
H3A	0.4505	0.7801	0.4356	0.031*	
H3B	0.3740	0.7191	0.3223	0.031*	
C4	0.4413 (2)	0.9955 (2)	0.34738 (15)	0.0396 (4)	
H4A	0.5295	1.0166	0.3991	0.059*	
H4B	0.4133	1.0834	0.3397	0.059*	
H4C	0.4612	0.9581	0.2848	0.059*	
N1	0.21672 (13)	0.70856 (13)	0.51881 (9)	0.0192 (2)	0.569 (2)
C5	0.12069 (19)	0.8002 (2)	0.52550 (12)	0.0312 (4)	0.569 (2)
H5A	0.1610	0.8662	0.5884	0.037*	0.569 (2)
H5B	0.1316	0.8599	0.4716	0.037*	0.569 (2)
C6	−0.0309 (3)	0.7431 (3)	0.5215 (2)	0.0246 (5)	0.569 (2)
H6A	−0.0480	0.6835	0.5749	0.030*	0.569 (2)
H6B	−0.0783	0.6824	0.4572	0.030*	0.569 (2)
O2	−0.0900 (2)	0.8591 (3)	0.53320 (18)	0.0365 (4)	0.569 (2)
C7	−0.2292 (3)	0.8147 (2)	0.50101 (19)	0.0547 (6)	0.569 (2)
H7A	−0.2693	0.8963	0.5028	0.082*	0.569 (2)
H7B	−0.2748	0.7471	0.5428	0.082*	0.569 (2)
H7C	−0.2499	0.7667	0.4329	0.082*	0.569 (2)
N1'	0.21672 (13)	0.70856 (13)	0.51881 (9)	0.0192 (2)	0.431 (2)
C5'	0.12069 (19)	0.8002 (2)	0.52550 (12)	0.0312 (4)	0.431 (2)
H5C	0.1118	0.8508	0.4671	0.037*	0.431 (2)
H5D	0.1553	0.8710	0.5863	0.037*	0.431 (2)
C6'	−0.0389 (4)	0.6765 (4)	0.5286 (3)	0.0246 (5)	0.431 (2)
H6C	−0.0856	0.6263	0.4611	0.030*	0.431 (2)
H6D	−0.0192	0.6046	0.5706	0.030*	0.431 (2)
O2'	−0.1349 (3)	0.7437 (3)	0.5672 (2)	0.0365 (4)	0.431 (2)
C7'	−0.2292 (3)	0.8147 (2)	0.50101 (19)	0.0547 (6)	0.431 (2)
H7D	−0.1720	0.8653	0.4560	0.082*	0.431 (2)
H7E	−0.2602	0.8830	0.5422	0.082*	0.431 (2)
H7F	−0.3159	0.7412	0.4625	0.082*	0.431 (2)
C8	0.29263 (16)	0.37698 (15)	0.78961 (10)	0.0188 (3)	
C9	0.14085 (17)	0.32430 (17)	0.75699 (11)	0.0238 (3)	
H9	0.0851	0.3895	0.7456	0.029*	
C10	0.07039 (18)	0.17834 (18)	0.74106 (12)	0.0278 (3)	
H10	−0.0327	0.1440	0.7177	0.033*	
C11	0.1507 (2)	0.08271 (17)	0.75925 (12)	0.0292 (3)	
H11	0.1028	−0.0174	0.7484	0.035*	
C12	0.3002 (2)	0.13313 (17)	0.79310 (13)	0.0294 (3)	
H12	0.3548	0.0674	0.8064	0.035*	
C13	0.37211 (17)	0.27965 (16)	0.80794 (11)	0.0236 (3)	
H13	0.4753	0.3132	0.8306	0.028*	
C14	0.32522 (16)	0.67665 (15)	0.94660 (10)	0.0194 (3)	
C15	0.23729 (18)	0.57911 (17)	0.99534 (12)	0.0259 (3)	
H15	0.2122	0.4795	0.9727	0.031*	
C16	0.1859 (2)	0.6256 (2)	1.07644 (13)	0.0328 (4)	
H16	0.1258	0.5579	1.1086	0.039*	
C17	0.22211 (19)	0.7699 (2)	1.11016 (12)	0.0321 (4)	
H17	0.1880	0.8017	1.1661	0.038*	
C18	0.30798 (19)	0.86851 (18)	1.06277 (12)	0.0287 (3)	
H18	0.3324	0.9680	1.0859	0.034*	
C19	0.35860 (17)	0.82221 (16)	0.98128 (11)	0.0233 (3)	
H19	0.4168	0.8907	0.9487	0.028*	
C20	0.62870 (16)	0.68280 (16)	0.84325 (11)	0.0214 (3)	
C21	0.71474 (19)	0.6659 (2)	0.77435 (13)	0.0339 (4)	
H21	0.6690	0.6197	0.7092	0.041*	
C22	0.8665 (2)	0.7157 (3)	0.79983 (15)	0.0433 (5)	
H22	0.9237	0.7029	0.7521	0.052*	
C23	0.93509 (19)	0.7839 (2)	0.89410 (14)	0.0373 (4)	
H23	1.0390	0.8178	0.9112	0.045*	
C24	0.85144 (19)	0.80232 (19)	0.96333 (13)	0.0316 (4)	
H24	0.8978	0.8494	1.0282	0.038*	
C25	0.69961 (17)	0.75194 (17)	0.93803 (12)	0.0246 (3)	
H25	0.6430	0.7648	0.9861	0.029*	

### (II) [Bis(2-methoxyethyl)dithiocarbamato-κ2S,S']triphenyltin(IV) Atomic displacement parameters (Å^2^)

**Table:**

	*U*^11^	*U*^22^	*U*^33^	*U*^12^	*U*^13^	*U*^23^
Sn	0.01785 (5)	0.01692 (5)	0.01786 (5)	0.00513 (4)	0.00269 (3)	0.00099 (3)
S1	0.02585 (18)	0.02457 (17)	0.01802 (16)	0.01231 (14)	0.00542 (13)	0.00314 (13)
S2	0.0303 (2)	0.02931 (19)	0.02422 (18)	0.01682 (16)	0.00519 (15)	0.00213 (14)
O1	0.0326 (6)	0.0268 (6)	0.0351 (6)	0.0064 (5)	0.0169 (5)	0.0082 (5)
C1	0.0189 (7)	0.0177 (6)	0.0196 (7)	0.0029 (5)	0.0037 (5)	0.0009 (5)
C2	0.0262 (7)	0.0245 (7)	0.0168 (7)	0.0056 (6)	0.0029 (5)	0.0002 (5)
C3	0.0256 (8)	0.0318 (8)	0.0222 (7)	0.0092 (6)	0.0080 (6)	0.0036 (6)
C4	0.0422 (11)	0.0335 (9)	0.0382 (10)	−0.0034 (8)	0.0184 (8)	0.0051 (8)
N1	0.0194 (6)	0.0211 (6)	0.0174 (6)	0.0059 (5)	0.0041 (4)	0.0027 (4)
C5	0.0347 (9)	0.0456 (10)	0.0231 (8)	0.0263 (8)	0.0067 (6)	0.0086 (7)
C6	0.0210 (9)	0.0262 (14)	0.0283 (10)	0.0093 (12)	0.0059 (7)	0.0029 (12)
O2	0.0243 (9)	0.0392 (9)	0.0445 (11)	0.0119 (8)	0.0048 (7)	−0.0067 (8)
C7	0.0675 (16)	0.0340 (11)	0.0552 (14)	0.0097 (10)	0.0002 (11)	0.0056 (10)
N1'	0.0194 (6)	0.0211 (6)	0.0174 (6)	0.0059 (5)	0.0041 (4)	0.0027 (4)
C5'	0.0347 (9)	0.0456 (10)	0.0231 (8)	0.0263 (8)	0.0067 (6)	0.0086 (7)
C6'	0.0210 (9)	0.0262 (14)	0.0283 (10)	0.0093 (12)	0.0059 (7)	0.0029 (12)
O2'	0.0243 (9)	0.0392 (9)	0.0445 (11)	0.0119 (8)	0.0048 (7)	−0.0067 (8)
C7'	0.0675 (16)	0.0340 (11)	0.0552 (14)	0.0097 (10)	0.0002 (11)	0.0056 (10)
C8	0.0220 (7)	0.0188 (6)	0.0155 (6)	0.0062 (5)	0.0038 (5)	0.0006 (5)
C9	0.0216 (7)	0.0254 (7)	0.0231 (7)	0.0065 (6)	0.0027 (5)	0.0006 (6)
C10	0.0235 (8)	0.0290 (8)	0.0254 (8)	0.0000 (6)	0.0058 (6)	−0.0021 (6)
C11	0.0390 (9)	0.0193 (7)	0.0273 (8)	0.0025 (6)	0.0133 (7)	−0.0003 (6)
C12	0.0383 (9)	0.0221 (7)	0.0330 (9)	0.0139 (7)	0.0127 (7)	0.0049 (6)
C13	0.0242 (7)	0.0239 (7)	0.0240 (7)	0.0094 (6)	0.0050 (6)	0.0026 (6)
C14	0.0196 (7)	0.0205 (6)	0.0177 (6)	0.0060 (5)	0.0028 (5)	0.0024 (5)
C15	0.0294 (8)	0.0217 (7)	0.0246 (7)	0.0037 (6)	0.0052 (6)	0.0050 (6)
C16	0.0325 (9)	0.0393 (9)	0.0264 (8)	0.0049 (7)	0.0126 (7)	0.0096 (7)
C17	0.0299 (9)	0.0455 (10)	0.0225 (8)	0.0127 (7)	0.0094 (6)	0.0002 (7)
C18	0.0330 (9)	0.0273 (8)	0.0258 (8)	0.0109 (7)	0.0056 (6)	−0.0037 (6)
C19	0.0247 (7)	0.0208 (7)	0.0231 (7)	0.0043 (6)	0.0064 (6)	0.0016 (5)
C20	0.0197 (7)	0.0213 (7)	0.0225 (7)	0.0054 (5)	0.0029 (5)	0.0040 (5)
C21	0.0242 (8)	0.0519 (11)	0.0231 (8)	0.0089 (7)	0.0044 (6)	−0.0002 (7)
C22	0.0242 (9)	0.0731 (15)	0.0324 (10)	0.0107 (9)	0.0105 (7)	0.0088 (9)
C23	0.0192 (8)	0.0489 (11)	0.0381 (10)	0.0011 (7)	0.0017 (7)	0.0124 (8)
C24	0.0270 (8)	0.0325 (8)	0.0289 (8)	0.0040 (7)	−0.0039 (6)	0.0029 (7)
C25	0.0257 (8)	0.0251 (7)	0.0223 (7)	0.0076 (6)	0.0029 (6)	0.0034 (6)

### (II) [Bis(2-methoxyethyl)dithiocarbamato-κ2S,S']triphenyltin(IV) Geometric parameters (Å, º)

**Table:**

Sn—C8	2.1312 (14)	C6'—H6D	0.9900
Sn—C20	2.1357 (15)	O2'—C7'	1.530 (4)
Sn—C14	2.1608 (14)	C7'—H7D	0.9800
Sn—S1	2.4612 (4)	C7'—H7E	0.9800
Sn—S2	3.0992 (4)	C7'—H7F	0.9800
S1—C1	1.7629 (14)	C8—C13	1.3945 (19)
S2—C1	1.6781 (14)	C8—C9	1.397 (2)
O1—C3	1.415 (2)	C9—C10	1.386 (2)
O1—C4	1.421 (2)	C9—H9	0.9500
C1—N1'	1.3340 (19)	C10—C11	1.386 (2)
C1—N1	1.3340 (19)	C10—H10	0.9500
C2—N1'	1.4712 (18)	C11—C12	1.379 (3)
C2—N1	1.4712 (18)	C11—H11	0.9500
C2—C3	1.509 (2)	C12—C13	1.394 (2)
C2—H2A	0.9900	C12—H12	0.9500
C2—H2B	0.9900	C13—H13	0.9500
C3—H3A	0.9900	C14—C19	1.395 (2)
C3—H3B	0.9900	C14—C15	1.397 (2)
C4—H4A	0.9800	C15—C16	1.390 (2)
C4—H4B	0.9800	C15—H15	0.9500
C4—H4C	0.9800	C16—C17	1.379 (3)
N1—C5	1.4651 (19)	C16—H16	0.9500
C5—C6	1.409 (3)	C17—C18	1.381 (3)
C5—H5A	0.9900	C17—H17	0.9500
C5—H5B	0.9900	C18—C19	1.390 (2)
C6—O2	1.414 (3)	C18—H18	0.9500
C6—H6A	0.9900	C19—H19	0.9500
C6—H6B	0.9900	C20—C21	1.393 (2)
O2—C7	1.284 (3)	C20—C25	1.395 (2)
C7—H7A	0.9800	C21—C22	1.387 (3)
C7—H7B	0.9800	C21—H21	0.9500
C7—H7C	0.9800	C22—C23	1.382 (3)
N1'—C5'	1.4651 (19)	C22—H22	0.9500
C5'—C6'	1.692 (5)	C23—C24	1.382 (3)
C5'—H5C	0.9900	C23—H23	0.9500
C5'—H5D	0.9900	C24—C25	1.388 (2)
C6'—O2'	1.419 (4)	C24—H24	0.9500
C6'—H6C	0.9900	C25—H25	0.9500
			
C8—Sn—C20	119.54 (5)	O2'—C6'—C5'	110.1 (3)
C8—Sn—C14	104.97 (5)	O2'—C6'—H6C	109.6
C20—Sn—C14	107.25 (6)	C5'—C6'—H6C	109.6
C8—Sn—S1	110.54 (4)	O2'—C6'—H6D	109.6
C20—Sn—S1	118.49 (4)	C5'—C6'—H6D	109.6
C14—Sn—S1	90.94 (4)	H6C—C6'—H6D	108.2
C8—Sn—S2	84.48 (4)	C6'—O2'—C7'	121.0 (3)
C20—Sn—S2	87.42 (4)	O2'—C7'—H7D	109.5
C14—Sn—S2	154.45 (4)	O2'—C7'—H7E	109.5
S1—Sn—S2	63.534 (11)	H7D—C7'—H7E	109.5
C1—S1—Sn	97.95 (5)	O2'—C7'—H7F	109.5
C1—S2—Sn	78.60 (5)	H7D—C7'—H7F	109.5
C3—O1—C4	111.94 (14)	H7E—C7'—H7F	109.5
N1'—C1—S2	123.54 (11)	C13—C8—C9	118.59 (14)
N1—C1—S2	123.54 (11)	C13—C8—Sn	121.76 (11)
N1'—C1—S1	116.67 (10)	C9—C8—Sn	119.58 (10)
N1—C1—S1	116.67 (10)	C10—C9—C8	121.01 (14)
S2—C1—S1	119.79 (9)	C10—C9—H9	119.5
N1'—C2—C3	112.94 (12)	C8—C9—H9	119.5
N1—C2—C3	112.94 (12)	C11—C10—C9	119.81 (15)
N1—C2—H2A	109.0	C11—C10—H10	120.1
C3—C2—H2A	109.0	C9—C10—H10	120.1
N1—C2—H2B	109.0	C12—C11—C10	119.86 (15)
C3—C2—H2B	109.0	C12—C11—H11	120.1
H2A—C2—H2B	107.8	C10—C11—H11	120.1
O1—C3—C2	108.64 (13)	C11—C12—C13	120.63 (15)
O1—C3—H3A	110.0	C11—C12—H12	119.7
C2—C3—H3A	110.0	C13—C12—H12	119.7
O1—C3—H3B	110.0	C8—C13—C12	120.07 (15)
C2—C3—H3B	110.0	C8—C13—H13	120.0
H3A—C3—H3B	108.3	C12—C13—H13	120.0
O1—C4—H4A	109.5	C19—C14—C15	117.90 (13)
O1—C4—H4B	109.5	C19—C14—Sn	121.67 (11)
H4A—C4—H4B	109.5	C15—C14—Sn	120.33 (10)
O1—C4—H4C	109.5	C16—C15—C14	121.04 (15)
H4A—C4—H4C	109.5	C16—C15—H15	119.5
H4B—C4—H4C	109.5	C14—C15—H15	119.5
C1—N1—C5	122.66 (12)	C17—C16—C15	119.95 (16)
C1—N1—C2	120.73 (12)	C17—C16—H16	120.0
C5—N1—C2	116.61 (12)	C15—C16—H16	120.0
C6—C5—N1	122.02 (19)	C16—C17—C18	120.08 (15)
C6—C5—H5A	106.8	C16—C17—H17	120.0
N1—C5—H5A	106.8	C18—C17—H17	120.0
C6—C5—H5B	106.8	C17—C18—C19	120.01 (15)
N1—C5—H5B	106.8	C17—C18—H18	120.0
H5A—C5—H5B	106.7	C19—C18—H18	120.0
C5—C6—O2	107.8 (2)	C18—C19—C14	121.00 (15)
C5—C6—H6A	110.1	C18—C19—H19	119.5
O2—C6—H6A	110.1	C14—C19—H19	119.5
C5—C6—H6B	110.1	C21—C20—C25	118.04 (14)
O2—C6—H6B	110.1	C21—C20—Sn	124.74 (12)
H6A—C6—H6B	108.5	C25—C20—Sn	117.17 (11)
C7—O2—C6	109.2 (2)	C22—C21—C20	120.73 (16)
O2—C7—H7A	109.5	C22—C21—H21	119.6
O2—C7—H7B	109.5	C20—C21—H21	119.6
H7A—C7—H7B	109.5	C23—C22—C21	120.52 (17)
O2—C7—H7C	109.5	C23—C22—H22	119.7
H7A—C7—H7C	109.5	C21—C22—H22	119.7
H7B—C7—H7C	109.5	C22—C23—C24	119.56 (16)
C1—N1'—C5'	122.66 (12)	C22—C23—H23	120.2
C1—N1'—C2	120.73 (12)	C24—C23—H23	120.2
C5'—N1'—C2	116.61 (12)	C23—C24—C25	119.97 (16)
N1'—C5'—C6'	100.59 (18)	C23—C24—H24	120.0
N1'—C5'—H5C	111.7	C25—C24—H24	120.0
C6'—C5'—H5C	111.7	C24—C25—C20	121.18 (15)
N1'—C5'—H5D	111.7	C24—C25—H25	119.4
C6'—C5'—H5D	111.7	C20—C25—H25	119.4
H5C—C5'—H5D	109.4		
			
Sn—S2—C1—N1'	−176.86 (13)	N1'—C5'—C6'—O2'	160.9 (3)
Sn—S2—C1—N1	−176.86 (13)	C5'—C6'—O2'—C7'	82.6 (3)
Sn—S2—C1—S1	3.05 (7)	C13—C8—C9—C10	−1.3 (2)
Sn—S1—C1—N1'	176.12 (10)	Sn—C8—C9—C10	−178.46 (12)
Sn—S1—C1—N1	176.12 (10)	C8—C9—C10—C11	1.1 (2)
Sn—S1—C1—S2	−3.80 (9)	C9—C10—C11—C12	0.0 (2)
C4—O1—C3—C2	177.33 (13)	C10—C11—C12—C13	−0.9 (3)
N1'—C2—C3—O1	−66.76 (16)	C9—C8—C13—C12	0.4 (2)
N1—C2—C3—O1	−66.76 (16)	Sn—C8—C13—C12	177.47 (12)
S2—C1—N1—C5	−177.49 (12)	C11—C12—C13—C8	0.7 (2)
S1—C1—N1—C5	2.6 (2)	C19—C14—C15—C16	−0.6 (2)
S2—C1—N1—C2	2.9 (2)	Sn—C14—C15—C16	−177.05 (13)
S1—C1—N1—C2	−177.05 (10)	C14—C15—C16—C17	−0.3 (3)
C3—C2—N1—C1	−90.53 (17)	C15—C16—C17—C18	0.8 (3)
C3—C2—N1—C5	89.80 (17)	C16—C17—C18—C19	−0.3 (3)
C1—N1—C5—C6	−90.6 (2)	C17—C18—C19—C14	−0.6 (2)
C2—N1—C5—C6	89.0 (2)	C15—C14—C19—C18	1.1 (2)
N1—C5—C6—O2	178.09 (18)	Sn—C14—C19—C18	177.44 (12)
C5—C6—O2—C7	160.8 (2)	C25—C20—C21—C22	0.4 (3)
S2—C1—N1'—C5'	−177.49 (12)	Sn—C20—C21—C22	−176.82 (16)
S1—C1—N1'—C5'	2.6 (2)	C20—C21—C22—C23	−0.3 (3)
S2—C1—N1'—C2	2.9 (2)	C21—C22—C23—C24	0.0 (3)
S1—C1—N1'—C2	−177.05 (10)	C22—C23—C24—C25	0.3 (3)
C3—C2—N1'—C1	−90.53 (17)	C23—C24—C25—C20	−0.2 (3)
C3—C2—N1'—C5'	89.80 (17)	C21—C20—C25—C24	−0.1 (2)
C1—N1'—C5'—C6'	−85.0 (2)	Sn—C20—C25—C24	177.30 (13)
C2—N1'—C5'—C6'	94.66 (19)		

### (II) [Bis(2-methoxyethyl)dithiocarbamato-κ2S,S']triphenyltin(IV) Hydrogen-bond geometry (Å, º)

Cg1 and Cg2 are the centroids of the C8–C13 and C14–C19 rings, respectively.

**Table:**

*D*—H···*A*	*D*—H	H···*A*	*D*···*A*	*D*—H···*A*
C7—H7*C*···*Cg*1^i^	0.98	2.94	3.821 (3)	151
C13—H13···*Cg*2^ii^	0.95	2.98	3.7979 (18)	145
C23—H23···*Cg*2^iii^	0.95	2.97	3.707 (2)	136

Symmetry codes: (i) −*x*, −*y*+1, −*z*+1; (ii) −*x*+1, −*y*+1, −*z*+2; (iii) *x*+1, *y*, *z*.
